# F131. CHILDHOOD ADVERSITIES IN PEOPLE AT ULTRA-HIGH RISK (UHR) FOR PSYCHOSIS: SYSTEMATIC REVIEW & META-ANALYSIS

**DOI:** 10.1093/schbul/sby017.662

**Published:** 2018-04-01

**Authors:** Oon Him Peh, Attilio Rapisarda, Jimmy Lee

**Affiliations:** Institute of Mental Health

## Abstract

**Background:**

Childhood adversities such as childhood abuse, bullying victimisation, and parental separation have been found to be associated with many psychiatric illnesses, including psychosis. A large body of research has been conducted on individuals at ultra-high risk (UHR) for psychosis, or clinical high risk (CHR) for psychosis. This review aims to quantitatively summarise (i) the associations between childhood adversities and the UHR state, and (ii) how these adversities may be linked with a higher risk of transition to psychosis (TTP).

**Methods:**

We conducted systematic searches based on PubMed, EMBASE, and PsycINFO databases. We adopted search terms aimed at retrieving studies related to: (1) populations which were at UHR of psychosis, (2) exposure to childhood adversities, and (3) schizophrenia. Studies were eligible as long as they reported information on any form of childhood adversities and recruited participants at UHR of psychosis. Studies that only investigated the level of psychotic symptoms in a cohort or among schizophrenia patients were excluded.

Whenever possible, we conducted meta-analyses to compare, across UHR and healthy individuals: (a) the levels of childhood trauma exposure, (b) childhood bullying victimisation, and (c) parental separation or loss. We conducted a second set of meta-analyses to investigate the effect of childhood trauma on TTP. Whenever allowed by provision of detailed information, we also conducted separate meta-analytic computations for each reported subtype of childhood adversity and trauma. All analyses were conducted in Review Manager 5.3, using inverse variance or Mantel-Haenszel methods (random effects model).

**Results:**

The systematic searches yielded 13 case-control, cross-sectional, and prospective studies from 27 publications, which recorded exposure to childhood adversities among UHR individuals: five of these studies employed longitudinal designs to investigate the conversion rate among UHR. Meta-analytic calculations revealed that, as compared to healthy controls, UHR individuals reported more severe childhood trauma (Random effects Hedges’ g = 1.38; 95% CI: 0.92–1.84, Z = 5.92, p < .001), were 5.5 times and 2.5 times more likely to report emotional abuse (OR = 5.54, 95% CI = 1.13–27.20, p = .03) and physical abuse (OR = 2.53, 95% CI = 0.73 - 8.76, p = .14) respectively. UHR individuals were 3.1 times as likely to report bullying victimisation (OR = 3.09, 95% CI = 2.23 - 4.30; Z = 6.72, p < .001). However, childhood trauma exposure in general was not significantly associated with psychotic conversion (HR = 1.01, 95% CI: 0.99 - 1.03; Z = 1.51, p = .13), suggesting perhaps that this risk is either mediated by other risk factors or that most specific traumatic experiences may contribute to an enhanced risk of conversion among UHR individuals.

**Discussion:**

To date, this is the first meta-analysis that quantitatively summarises the associations between childhood adversities and TTP, and between specific abuse subtypes and the UHR state or TPP. Overall, our findings support the association between childhood adversities (trauma and bullying) and the UHR state; however, these adversities alone may not be sufficient to cause a UHR individual to develop frank psychosis. Most studies did not adjust for potentially confounding variables such as cannabis use, gender, education level, age, comorbid psychiatric disorders and other unmeasured variables such as socioeconomic status, urbanicity, genetic risk, and PTSD symptoms. The current review supports the need to screen for childhood adversities among the UHR population and to provide treatment accordingly, which may improve patients’ engagement with their treatments and result in better clinical outcomes.

